# Auxin production in diploid microsporocytes is necessary and sufficient for early stages of pollen development

**DOI:** 10.1371/journal.pgen.1007397

**Published:** 2018-05-29

**Authors:** Xiaozhen Yao, Lei Tian, Jun Yang, Yan-Na Zhao, Ying-Xiu Zhu, Xinhua Dai, Yunde Zhao, Zhong-Nan Yang

**Affiliations:** 1 College of Life and Environment Sciences, Shanghai Normal University, Shanghai, China; 2 Sciences Center for Excellence in Molecular Plant Sciences, Institute of Plant Physiology and Ecology, Chinese Academy of Sciences, Shanghai, China; 3 Section of Cell and Developmental Biology, University of California San Diego, La Jolla, California, United States of America; Fudan University, CHINA

## Abstract

Gametophytic development in Arabidopsis depends on nutrients and cell wall materials from sporophytic cells. However, it is not clear whether hormones and signaling molecules from sporophytic tissues are also required for gametophytic development. Herein, we show that auxin produced by the flavin monooxygenases YUC2 and YUC6 in the sporophytic microsporocytes is essential for early stages of pollen development. The first asymmetric mitotic division (PMI) of haploid microspores is the earliest event in male gametophyte development. Microspore development in *yuc2yuc6* double mutants arrests before PMI and consequently *yuc2yuc6* fail to produce viable pollens. Our genetic analyses reveal that *YUC2* and *YUC6* act as sporophytic genes for pollen formation. We further show that ectopic production of auxin in tapetum, which provides nutrients for pollen development, fails to rescue the sterile phenotypes of *yuc2yuc6*. In contrast, production of auxin in either microsporocytes or microspores rescued the defects of pollen development in *yuc2yuc6* double mutants. Our results demonstrate that local auxin biosynthesis in sporophytic microsporocytic cells and microspore controls male gametophyte development during the generation transition from sporophyte to male gametophyte.

## Author summary

Plant life cycle alternates between the diploid sporophyte generation and the haploid gametophyte generation. Understanding the molecular mechanisms governing the generation alternation impacts fundamental plant biology and plant breeding. It is known that the development of haploid generation in vascular plants requires the diploid tapetum cells to supply nutrients. Here we show that the male gametophyte (haploid) development in Arabidopsis requires auxin produced in the diploid microsporocytic cells. Moreover, we show that auxin produced in microsporocytic cells and microspore is also sufficient to support normal development of the haploid microspores. This work demonstrates that Arabidopsis uses two different diploid cell types to supply growth hormone and nutrients for the growth of the haploid generation.

## Introduction

Life cycle of eukaryotes alternates between haploid and diploid generations. The alternation of generations is initiated by meiosis (2n to 1n) and gamete fusion (1n to 2n) [1]. In land plants, the multicellular diploid generation is called sporophyte, whereas the multicellular haploid organism is named gametophyte. In bryophytes (mosses and liverworts), haploid gametophyte is the dominant generation and represents the main plant. In vascular plants, including ferns, gymnosperms, and angiosperms, the diploid sporophyte generation is dominant, whereas the gametophyte generation is much reduced [1]. For example, in seed plants, both the female and male gametophytes develop within the sporophyte. Understanding the molecular mechanisms governing the generation alternation will impact fundamental plant biology and plant breeding.

Pollen grains, which are the male gametophyte in seed plants, are developed in locules encircled by four sporophytic cell layers: tapetum, middle layer, endothecium, and epidermis. Inside a locule, a diploid male meiocyte divides into a tetrad of four haploid microspores after meiosis [2, 3]. Each microspore then undergoes an asymmetric cell division (pollen mitosis I (PMI)), resulting in two structurally and functionally different daughter cells: the small generative cell and the large vegetative cell. The generative cell divides one more time (PMII) to produce two sperm cells whereas the vegetative cell no longer divides. The mature pollen grain contains two haploid sperm cells and one haploid vegetative cell [3, 4].

Genetic analyses have identified a number of genes that play important roles in pollen development [5]. These genes can be classified into two categories: gametophytic or sporophytic genes. Pollen development depends on coordinated expression of both sporophytic genes and gametophytic genes [3]. Most of the identified gametophytic genes are related to cell division and differentiation. For example, MOR1, a member of the microtubule-associated protein 215 (MAP215), is involved in the PMI asymmetric cell division [6, 7]. In the *mor1/gem1* mutants, defects in microspore nucleus migration lead to altered division plane, and the formation of two equal- or similar sized cells [6, 7]. Genes including *Two-in-one* (*TIO*), *GAMMA-TUBULIN 1* (*TUBG1*) and *2* essential for phragmoplast formation, localization and/or expansion, also affect male gametophyte development. Mutations in these genes cause incomplete cytokinesis in PMI and result in failure to produce the generative cell [8–13]. Several cell cycle factors have also been reported to be important for pollen mitosis. ICK4/KRP6 (Interactors of Cdc2 kinase 4/kip-related protein 6) is a cyclin-dependent kinase inhibitor. Timely degradation of ICK6/KRP6 by RHF1a/2a and SCF^FBL17^ E3 ligase is essential for the progression of pollen mitosis [14–16]. Cyclin-dependent kinase D (CDKD) was recently found to be essential for pollen mitosis. In the *cdkd;1–1 cdkd;3–1* double mutants, microspore is defective in both PMI and PMII [17]. A main feature of the male gametophytic genes is that no viable mutant pollens can be generated and that no homozygous diploid mutants are available.

Sporophytic genes for pollen formation represent the contribution of sporophytic tissues including tapetum and microsporocyte for male gametophyte development. Tapetum directly provides pollen wall materials and nutrients including Magnesium for pollen development [18–22]. A series of transcription factors including DYT1, TDF1, AMS, MYB33/65, MS188/MYB80/MYB103, and MS1 have been shown to play essential roles in normal development of tapetum [23–32]. The defective tapetum caused by mutations in these genes results in pollen wall defects and leads to complete pollen abortion [23–32]. Enzymes involved in outer pollen wall formation are also expressed in tapetum and are essential for pollen development [33–41]. The pollen wall pattern is determined inside a tetrad that depends on the sporophytic genes expressed in microsporocytes, such as *RPG1*, *NPU*, *NEF* and *DEX1* [42–45]. The sporophytic cells including tapetum and microsporocyte supply material and nutrients for pollen development and determine the pollen wall pattern. Unlike gametophytic genes, viable mutant pollen can be produced from heterozygous mutant plants, and homozygous mutant plants can be obtained. However, homozygous diploid mutants cannot produce viable pollens.

Plant hormones are essential for normal plant development. However, it is not clear whether plant hormones or other signaling molecules produced in sporophytic tissues are required for the development of male gametophyte. The plant hormone auxin plays critical roles in nearly all aspects of plant development including embryogenesis, organogenesis, gametophyte development, and various tropisms [46]. It is known that disruption of either auxin biosynthesis, or polar transport, or signaling causes defects in male gametophyte development and pollen formation. Indole-3-acetic acid (IAA), the primary natural auxin in plants, is mainly synthesized through a TAA/YUC two-step tryptophan-dependent pathway [47–51]. Simultaneously disruption of both *YUC2* and *YUC6* completely eliminates the production of viable pollen grains [49]. It was reported that the two atypical members of the PIN auxin efflux carriers, PIN5 and PIN8, which are believed to regulate intracellular auxin homeostasis and metabolism in pollen, also participate in the development of normal pollen morphology [52, 53]. Other auxin transporters including ATP-BINDING CASSETTE B19 (ABCB19)/MULTIDRUG RESISTANCE PROTEIN 1 (MDR1)/ P-GLYCOPROTEIN 19 (PGP19) and ABCB1/ PGP1 also play important roles in pollen development [54, 55]. The *abcb1 abcb19* double mutants show precocious pollen maturation [54, 55]. Similar precocious pollen maturation takes place in *tir1 afb2 afb3* triple and *tir1 afb1 afb2 afb3* quadruple mutants, which are defective in auxin perception [54]. It is known that auxin biosynthetic, transport, and signaling genes are expressed during pollen development [49, 54].

Previous studies have clearly demonstrated that auxin is required for anther development and pollen formation [49, 52–55], but it was not understood the role of auxin for pollen development and in the transition from sporophytic generation to gametophyte generation. It was previously proposed that auxin produced in tapetum is required for pollen development [56]. Here we report that pollen development in the auxin biosynthetic mutants *yuc2yuc6* failed to progress past the PMI, which is an early step in male gametophyte growth. Our genetic analyses demonstrated that both *YUC2* and *YUC6* function as sporophytic genes. We further show that auxin produced in sporophytic microsporocytes rather than tapetum is required for the early stages of pollen development, demonstrating that auxin produced in the diploid sporophytic cells plays a critical role in the haploid male gametophyte development.

## Results

### YUC2 and YUC6 are the main auxin biosynthetic flavin monooxygenases in anther

**Fig 1:**
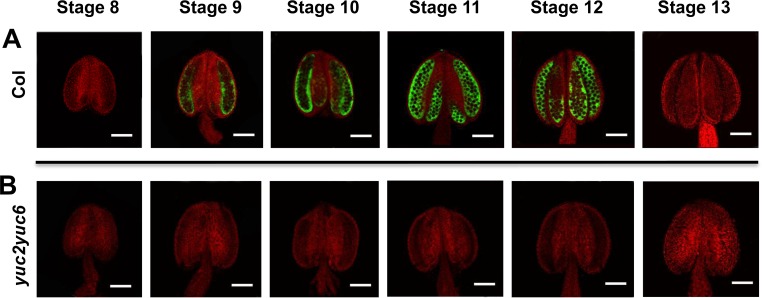
The expression patterns of DR5:GFP in the anther of *yuc2yuc6*. (A-B) Comparison of the auxin reporter *DR5*:*GFP* signal in anthers of wild type (A) and *yuc2yuc6* (B) at different developmental stages. Reporter signal in anthers observed in wild type from stages 9 to 12 is completely absent in *yuc2yuc6*. Bars = 100 μm.

We previously showed that *yuc2yuc6* double mutants were male sterile, and the expression of the bacterial auxin biosynthetic gene *iaaM* under the control of the *YUC6* promoter fully restored the fertility of *yuc2yuc6*, indicating that the fertility defects of *yuc2yuc6* were caused by partial auxin deficiency during anther development [49]. The auxin reporter *DR5-GFP/GUS* has been previously detected in anther from flower stages 10 to 12 [54, 57, 58]. It has also been reported that the DR5 activity is decreased in the *yuc6* single mutant, although *yuc6* did not display obvious reproductive defect [58]. To better understand the distribution patterns of DR5 in anthers of *yuc2yuc6*, we introduced the auxin reporter DR5-GFP into *yuc2yuc6* plants and compared the GFP signals in *yuc2yuc6* with those in WT plants. Consistent with previous findings [54, 57, 58], GFP signals in WT plants were detected in anthers from anther stages 9 to 12 (all the stages refer to anther stages in our results) (Fig 1A). The expression pattern of DR5-GFP in *yuc2* and *yuc6* anthers was similar to that in WT (S1 Fig). However, no substantial signals were detected in *yuc2yuc6* anthers at the same developmental stages (Fig 1B). We also introduced the *DR5-GUS* into the *yuc2yuc6* background. Similar to the DR5-GFP patterns, GUS signals were not detected in the *yuc2yuc6* anthers (S1 Fig). Therefore, it appears that *YUC2* and *YUC6* are the main auxin biosynthetic genes responsible for auxin production during anther development.

### Expression of auxin reporters in unicellular microspores

**Fig 2:**
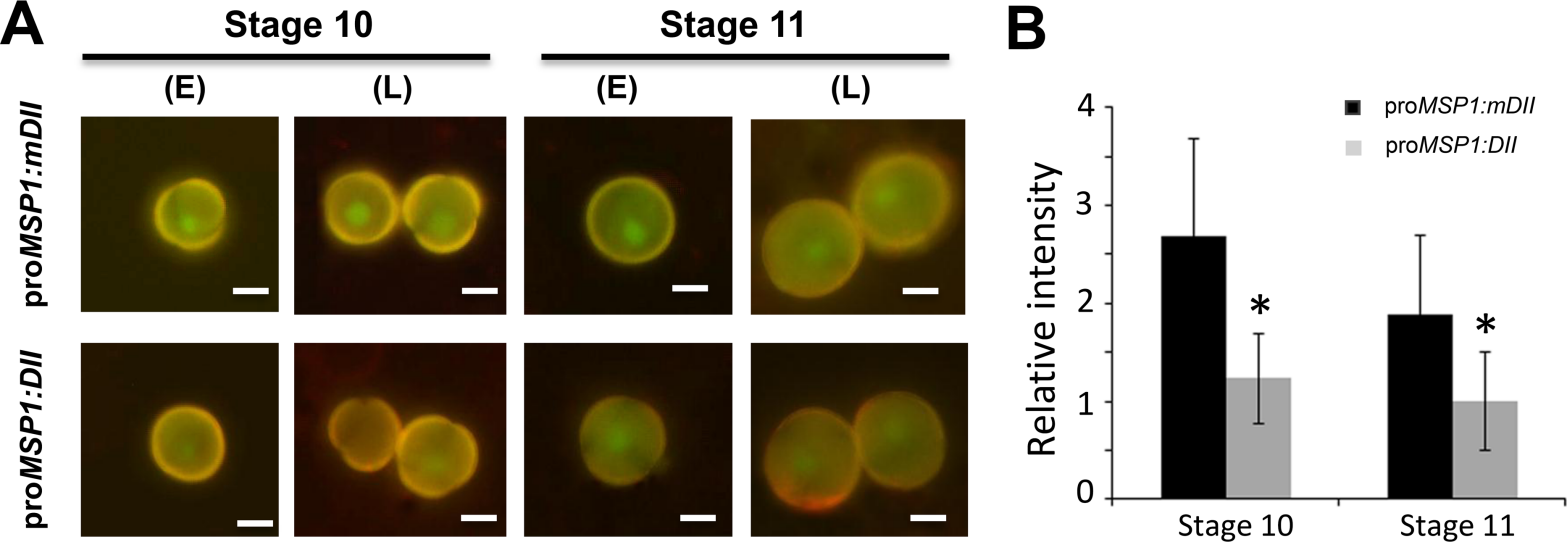
DII-VENUS for visualizing auxin-induced Aux/IAA degradation during pollen development. (A) Fluorescence of pro*MSP1*:*mDII* control and pro*MSP1*:*DII* plants. *MSP1* promoter (pro*MSP1*) is a microspore-specific promoter. E: early, L: late. (B) Quantitative representation of the relative VENUS fluorescence intensity from nucleus of stage 10 and stage 11 pollen. The fluorescence intensity was measured using ImageJ software. Data shown in B represent mean with SD based on more than 20 pollen grains for each group (set as 1 for stage 11 pollen from pro*MSP1*:*DII*). The symbol * indicates where the difference between pro*MSP1*:*DII* and pro*MSP1*:*mDII* at corresponding stage is significant in statistic test (*p*-value<0.05). Note that the fluorescence signals of DII in stage 10 and stage 11 were much weaker than that in mDII microspores. Bars = 7 μm.

Because we hardly observed any DR5-GFP signal in pollen, we used DII and mDII auxin reporter lines as a proxy to determine the pattern of auxin-induced degradation of Aux/IAA repressors during pollen development [59]. We replaced the 35S promoter with the microspore-specific promoter pro*MSP1* to drive the *DII-VENUS* and *mDII-VENUS* expression units [60]. Our results showed that fluorescence signals in microspores/pollens of pro*MSP1*:*DII-VENUS* transgenic plants were weaker than that in pro*MSP1*:*mDII-VENUS* transgenic plants at stage 10 (pro*MSP1*:*DII-VENUS/*pro*MSP1*:*mDII-VENUS = 0*.*45*) and at stage 11 (pro*MSP1*:*DII-VENUS/*pro*MSP1*:*mDII-VENUS = 0*.*54*)) (Fig 2A and 2B). The results of the auxin response reporters are indicative that auxin accumulated significantly in unicellular microspores and bicellular pollens.

To investigate whether signals of the auxin response reporters are correlated with the expression patterns of *YUC2* and *YUC6*, we generated pro*YUC2-GFP* and pro*YUC6-GFP* transgenic plants. We found that both *YUC2* and *YUC6* were weakly expressed in microsporocytes, tetrad and microspores at stage 9 (S2A, S2B, S2C, S2I, S2J and S2O Fig) and strongly expressed in microspores from stages 10 to 13 (S2D–S2G and S2K–S2N Fig). We also found that *YUC2* and *YUC6* were strongly expressed in somatic cell layers including tapetum cells in anther (S2H, S2I and S2O Fig).

### Early stages including pollen mitosis I (PMI) are defective in *yuc2yuc6*

**Fig 3:**
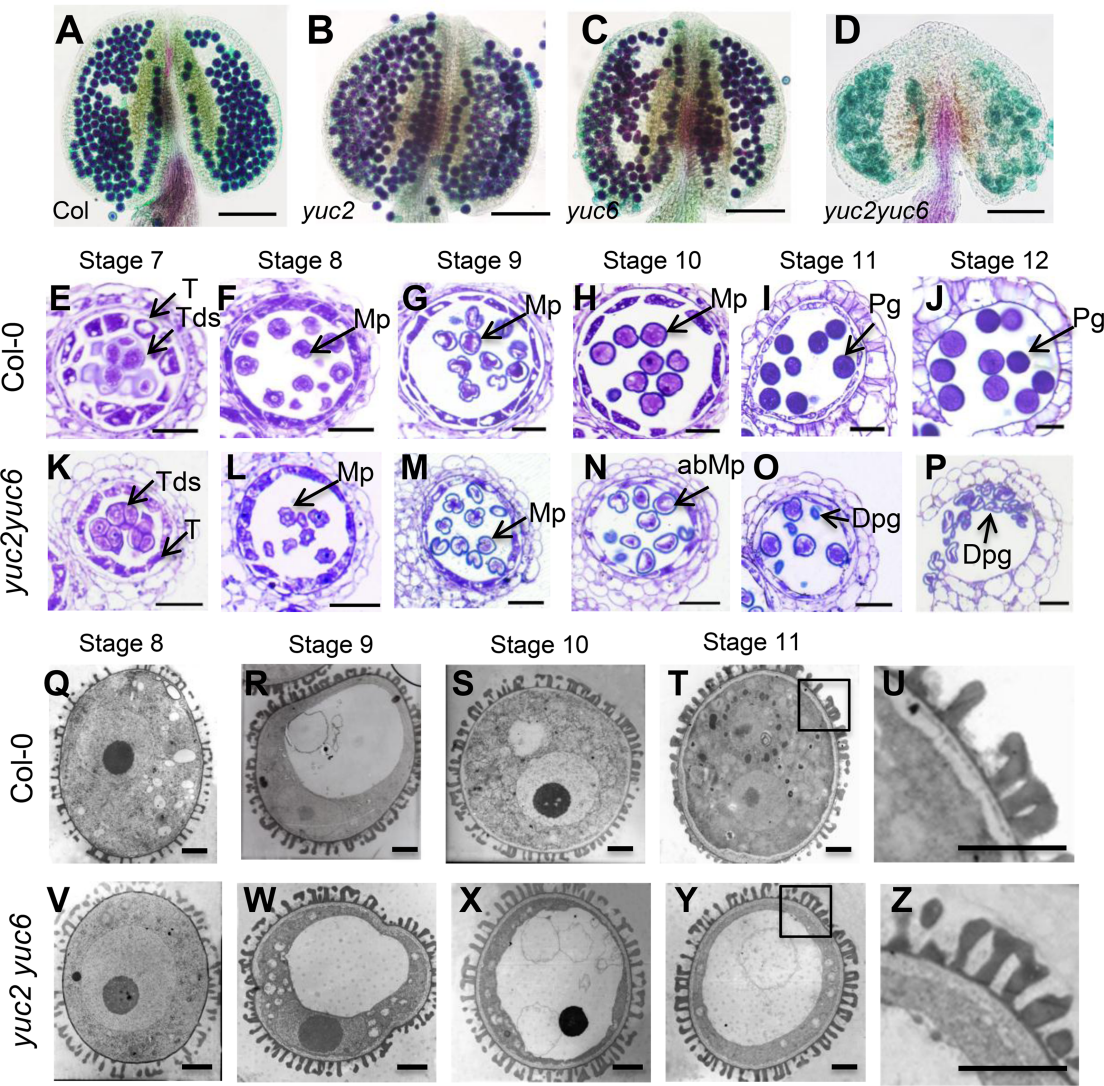
The *yuc2yuc6* double mutants were defective in pollen development. (A-D) Alexander staining of anthers of wild type (A), *yuc2* (B), *yuc6* (C) and *yuc2yuc6* (D). Note that *yuc2yuc6* lacked viable pollen. (E-P) Semi-thin cross sections of anthers from wild type (E-J) and *yuc2yuc6* (K-P) were stained with toluidine blue. Anthers at (E and K) stage 7; (F and L) stage 8; (G and M) stage 9; (H and N) stage 10; (I and O) stage 11; (J and P) stage 12. A microspore or pollen showing reduced or completely invisible of cytoplasm we defined as degeneration state. Note that the degenerated microspores were evident in *yuc2yuc6* from stage 10 to 12. (Q-Z) Ultra-structures of pollen grains from wild type (Q-U) and *yuc2yuc6* (V-Z) plants. Microspores from anthers at stage 8 (Q and V); stage 9 (R and W); stage 10 (S and X); stage 11 (T and Y) were shown. Enlarged images from T and Y were shown in (U and Z**)**. It is clear that the pollen wall pattern in *yuc2yuc6* appears similar to that in WT. Most of the cytoplasm is degenerated in the microspore from stage 10 and 11 anthers of *yuc2yuc6* (X and Y). T, Tapetum; Tds, tetrads; Mp, microspore; abMp, abnormal microspore; Pg, pollen grain; Dpg, degenerated pollen grain. Bars = 100μm (A-D), 20 μm (E-P), 1 μm (Q-Z).

The *yuc2yuc6* double mutants showed markedly reduced fertility with few viable pollen inside the locule [49, 54]. Pollination of the *yuc2yuc6* pistil with WT pollen resulted in F1 plants with normal fertility (~50 seeds produced in each silique, n = 4), indicating that the female fertility of *yuc2yuc6* was unaffected. Alexander staining was performed to understand the defects of pollen development in *yuc2yuc6*. Similar to those in wild type (WT), the anthers of *yuc2* and *yuc6* single mutants contained purple-stained viable pollen grains (Fig 3A–3C). Consistent with previous reports [49], Fig 3D showed that the *yuc2yuc6* anthers did not produce viable pollens (Fig 3D). We then generated anther cross sections and used transmission electron microscopy to determine in which stage(s) the anther and pollen developmental defects took place in *yuc2yuc6*. We noticed that a normal tetrad was produced in *yuc2yuc6* (Fig 3E and 3K), suggesting that the meiotic division progressed normally. After release from the tetrad, microspore development in *yuc2yuc6* appeared similar to that of WT from stages 8 to 9 (Fig 3F, 3G, 3L, 3M, 3Q, 3R, 3V and 3W). However, at stage 10, WT microspores became vacuolated and contained a polarized nucleus (Fig 3H and 3S), whereas *yuc2yuc6* microspores had an irregular shape and started to degenerate (Fig 3N and 3X). From stages 11 to 12, WT microspores underwent the first mitotic division and gradually developed into mature pollen (Fig 3I, 3J and 3T). In *yuc2yuc6*, microspores were severely degenerated and failed to form normal pollen (Fig 3O, 3P and 3Y). The pollen wall of *yuc2yuc6* appeared normal as compared with that of WT (Fig 3U and 3Z).

**Fig 4:**
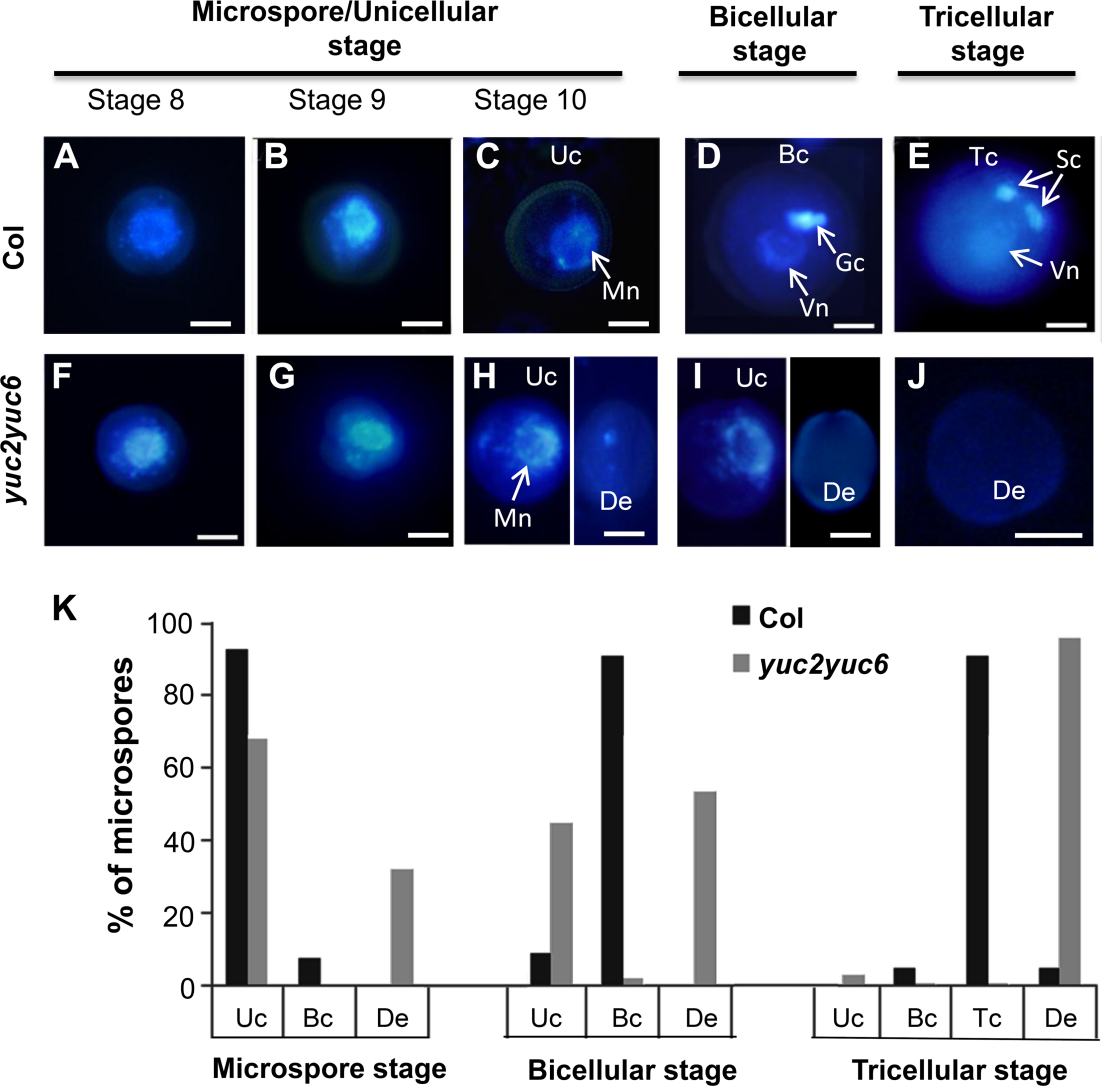
The early stages of male gametogenesis were defective in the *yuc2yuc6* double mutants. (A-J) DAPI staining of pollens from wild type (A-E) and *yuc2yuc6* (F-J) at different developmental stages. Microspores from anthers at (A and F) stage 8; (B and G) stage 9; (C and H) stage 10 were shown. Some of the *yuc2yuc6* microspores were degenerated and had little DAPI staining (H right). (D and I) pollens at the bicellular stage. Note that the nuclei of *yuc2yuc6* were arrested at the unicellular state or degenerated and could not form two nuclei as in the wild type (I). (E and J) Pollen at the tricellular stage. The *yuc2yuc6* pollen grains lacked genomic DNA (J) while WT pollen showed strong DAPI staining (E). (K) Quantitative analysis and comparison of pollen defects between wild type and *yuc2yuc6* (n>600 for each stage). Uc, Unicellular pollen; Mn, microspore nucleus; Gc, generative cell; Vn, vegetative nucleus; Sc, sperm cell. Bc, Bicellular pollen; Tc, Tricellular pollen; De, Degenerated pollen. Bars = 5 μm.

To obtain further insight into the microgametogenesis defects of *yuc2yuc6*, we stained nuclei with 4’, 6-diamidino-2-phenylindole (DAPI) in developing pollen. The microspores of *yuc2yuc6* were similar to WT microspores at stages 8 to 9 (Fig 4A, 4B, 4F and 4G). However, at stage 10, some of the *yuc2yuc6* microspores were degenerated, with little DAPI staining signal (Fig 4H right). At this stage, some normal *yuc2yuc6* microspores with a nucleus located at one side of the microspore were still visible (Fig 4C and 4H left). Overall during the unicellular stage (from stages 8 to 10), most of the single haploid cells in both the WT (92.7%) and *yuc2yuc6* (67.9%) showed a bright nucleus (Fig 4A–4C, 4F–4H and 4K), and about 30% of the microspores in *yuc2yuc6* were degenerated (Fig 4H right, 4K). After PMI, 91.1% of the WT microspores divided asymmetrically, producing a large vegetative cell and a small generative cell engulfed in the cytoplasm of the vegetative cell (Fig 4D and 4K), which is called the bicellular stage. In contrast, only about 2.2% of the *yuc2yuc6* microspores progressed past PMI to reach the bicellular stage (Fig 4K). Approximately 44.8% of the microspores in *yuc2yuc6* remain arrested at the unicellular stage (Fig 4I left, 4K), and the rest (53%) became degenerated (Fig 4I right, 4K). At the tricellular stage, WT microspores fully developed into tricellular pollen, whereas *yuc2yuc6* microspores (96%) became completely degenerated or still contained a very loosely packed DNA mass (Fig 4E, 4J and 4K). Therefore, we conclude that mutations in *YUC2* and *YUC6* lead to the defects in early stages including PMI of pollen development.

We performed genetic complementation to determine whether mutations in the *YUC* genes are responsible for the sterile phenotype of *yuc2yuc6*. The DNA fragment including about 2 kb upstream sequence of *YUC2* and the *YUC2* open reading frame (ORF) was fused with *GFP* (named pro*YUC2*:*YUC2-GFP*) and the construct was introduced into *yuc2*^*-/-*^ *yuc6*^*+/-*^ plants (S3 Fig). Two independent lines of *yuc2yuc6* that contained the pro*YUC2*:*YUC2-GFP* transgene were identified in T1 generation. Both lines of pro*YUC2*:*YUC2-GFP* in the *yuc2yuc6* background had normal fertility (S3B Fig). Alexander staining and DAPI staining results indicated that *YUC2* could rescue the pollen development defects of *yuc2yuc6* (S3C and S3D Fig and S5 Fig). The results also demonstrated that the YUC2-GFP fusion is functional.

### Auxin produced in sporophytic cells controls gametophyte development

From our phenotypic analysis, it was clear that the *yuc2yuc6* double mutants were defective in gametophyte development. Gametophyte development depends on the expression of both sporophytic and gametophytic genes. To determine whether *YUC2* and *YUC6* function as sporophytic or gametophytic genes, we analyzed the male transmission efficiency of *yuc2yuc6*. We used *yuc2*^*-/-*^*yuc6*^*+/-*^ and *yuc2*^*+/-*^*yuc6*^*-/-*^ plants as pollen donors to cross with WT plants. PCR genotyping revealed that approximately 50% of F1 progeny contained the *yuc2yuc6* mutations (Table 1), suggesting that *yuc2yuc6* microspores were transmitted normally. We next analyzed the segregation ratio of self-fertilized *yuc2*^*-/-*^*yuc6*^*+/-*^and *yuc2*^*+/-*^*yuc6*^*-/-*^ plants. Consistent with the normal transmission efficiency, the segregation displayed a typical Mendelian ratio (3:1) in both cases (n>290 for each case). These results showed that *YUC2* and *YUC6* are sporophytic genes for pollen development, indicating that auxin produced in the sporophytic tissues by the YUCs is required for normal male gametophyte development.

**Table 1:** Effects of mutations in *YUC2* and *YUC6* on male transmission frequency.

Parental Genotype (Male × Female)	Progeny				total	TE	χ^2^
	*yuc2* ^*+/-*^	*yuc2* ^*+/+*^	*yuc6* ^*+/-*^	*yuc6* ^*+/+*^			
*yuc2* ^*-*/-^ *yuc6* ^+/-^ × WT	172		84	88	172	95.50%	0.093 (p>0.05)
*yuc2* ^+/-^ *yuc6* ^*-/-*^ × WT	91	86	177		177	105.80%	0.141 (p>0.05)

Transmission efficiencies (TE) = number of progenies with T-DNA insertion/number of progenies without T-DNA insertion. Expected values were based on the prediction that if the double mutant alleles were transmitted normally, about 50% of the progeny should receive the *yuc2yuc6* knockout allele from the *yuc2*^*-/-*^ *yuc6*^*+/-*^ or *yuc2*^*-/+*^ *yuc6*^*-/-*^ parents.

### Ectopic expression of *YUC2* in tapetum did not rescue the defects of pollen development in *yuc2yuc6*

**Fig 5:**
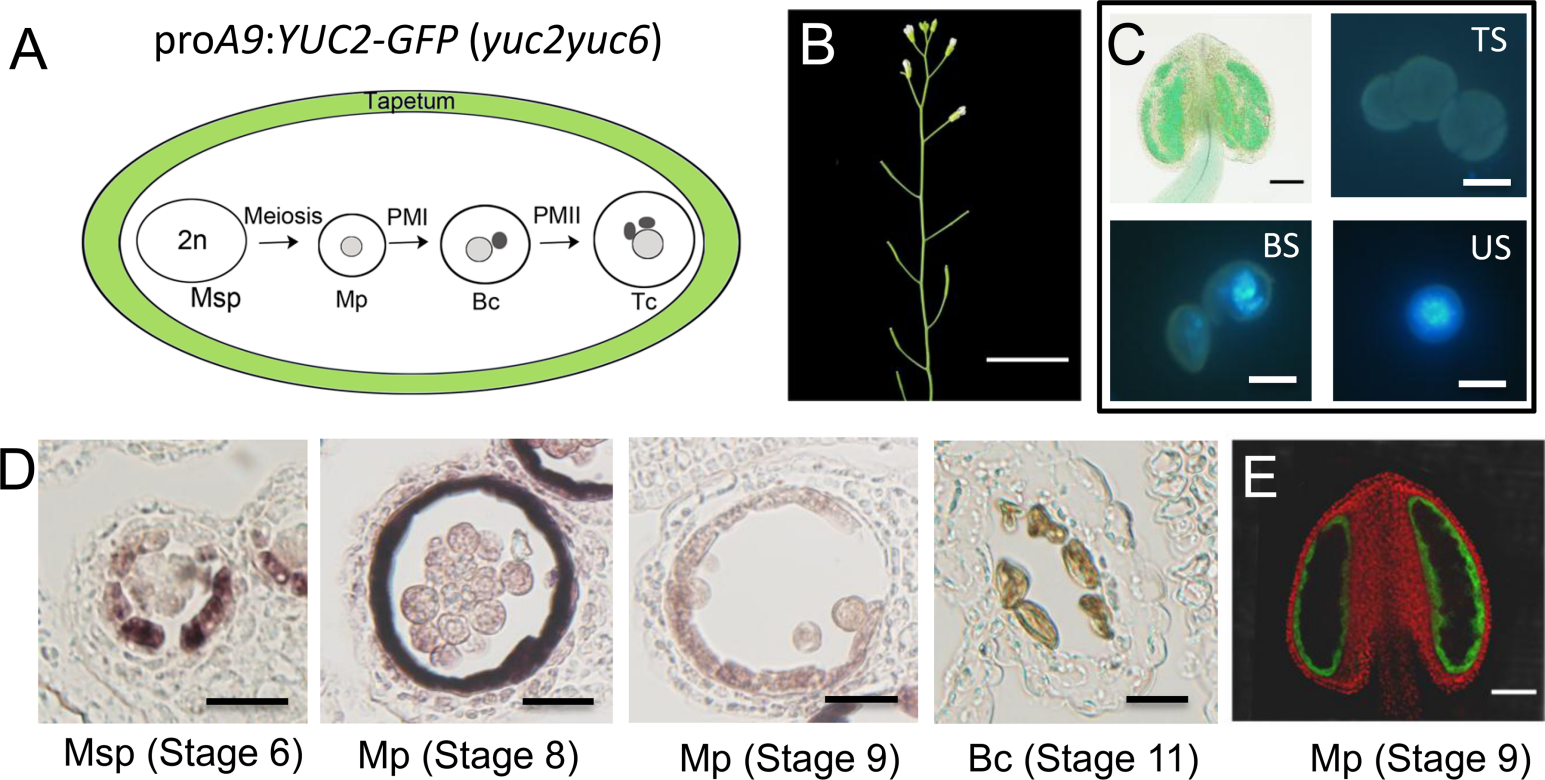
Localized auxin production in tapetum did not rescue the pollen defects in *yuc2yuc6* mutants. (A) The green color indicates the expression pattern of the *A9* promoter. Msp, Microsporocytes; Mp, microspores; Bc, Bicellular pollen; Tc, Tricellular pollen. The *A9* promoter cloned from Arabidopsis is used to drive *YUC2-GFP* expression specifically in tapetum cells from stages 6 to 9 [61, 62]. (B) Morphology of adult shoots (Bars = 2cm). (C) Alexander staining (Bars = 100 μm) and DAPI staining (Bars = 10 μm) of Pro*A9*:*YUC2-GFP* (*yuc2yuc6*) anthers and pollens. Note that the sterility phenotype and pollen defects were not rescued in Pro*A9*:*YUC2-GFP* (*yuc2yuc6*) transgenic plants. TS, Tricellular Stage; BS, Bicellular Stage; US; Unicellular Stage. (D) In situ hybridization of GFP in Pro*A9*:*YUC2-GFP* (*yuc2yuc6*) transgenic plants (Bars = 20 μm). (E) Fluorescence images (Bars = 100 μm) of the YUC2-GFP fusion protein in anthers from *yuc2yuc6* transformed with Pro*A9*:*YUC2-GFP*. In Pro*A9*: *YUC2-GFP* anther, GFP is significantly expressed in tapetum cell of microsporocyte stage and early microspore stage.

It is known that *YUC2/6* mRNA is expressed in meiocytes, microspores, tapetum, middle layer, and endothecium in anthers [54]. To investigate the source of auxin for pollen development, we used various promoters to drive the expression of YUC2-GFP fusion, which we have shown functional (S3 Fig). The DR5 auxin reporter line showed an extremely active auxin response in the tapetum cells during late developmental stages in Arabidopsis [54, 56, 58]. It was proposed that auxin is transported from tapetum cells into developing pollens [56]. To investigate whether the *YUC* genes expressed in tapetum are responsible for regulating microspore development and pollen formation, we generated transgenic lines that express *YUC2-GFP* in tapetum cells using specific tapetum promoters (pro*A9* and pro*ATA7* (*ARABIDOPSIS THALIANA ANTHER7*)) in the *yuc2yuc6* background [32, 61, 62] (Fig 5 and S4 Fig). We found that both pro*A9*: *YUC2-GFP* (*yuc2yuc6*) (n = 6) and pro*ATA7*:*YUC2-GFP* (*yuc2yuc6*) (n = 7) T1 transgenic plants were still sterile (Fig 5B and S4 Fig). The pollen defects in *yuc2yuc6* were not rescued by the *YUC2-GFP* transgene (Fig 5C, S4 and S5 Figs). We investigated whether the tapetum-specific promoters behaved as designed. RNA in situ hybridization data showed that *YUC2-GFP* was significantly transcribed in tapetum cells from microsporocytes stage to early microspore stage in pro*A9*:*YUC2-GFP* (*yuc2yuc6*) plants (Fig 5D). The GFP signal in pro*A9*:*YUC2-GFP* (*yuc2yuc6*) plants appeared in the tapetum layer at stages 8 and 9 (Fig 5E showed stage 9). Although the GFP fluorescence of pro*ATA7*:*YUC2-GFP* (*yuc2yuc6*) plants could not be detected (S4 Fig), the *YUC2-GFP* transcripts were detected in tapetum layer at stage 8 (S4 Fig). To rule out the possibility that the *ATA7* promoter was too weak to drive adequate expression of *YUC2-GFP* in anther, we used real-time quantitative RT-PCR to analyze the transcript levels of *YUC2* in WT and *YUC2-GFP* in the transgenic plants. The expression levels of *YUC2-GFP* in all of the analyzed transgenic plants were similar to or higher than *YUC2* expression in WT (S5 Fig). These results suggest that the *ATA7* and A9 promoters were able to drive *YUC2-GFP* expression in tapetum, but auxin production in tapetum is not sufficient to overcome the auxin deficiency in *yuc2yuc6* microspores.

### Expression of *YUC2-GFP* in microsporocyte and microspores rescued the defects of pollen development in *yuc2yuc6*

**Fig 6:**
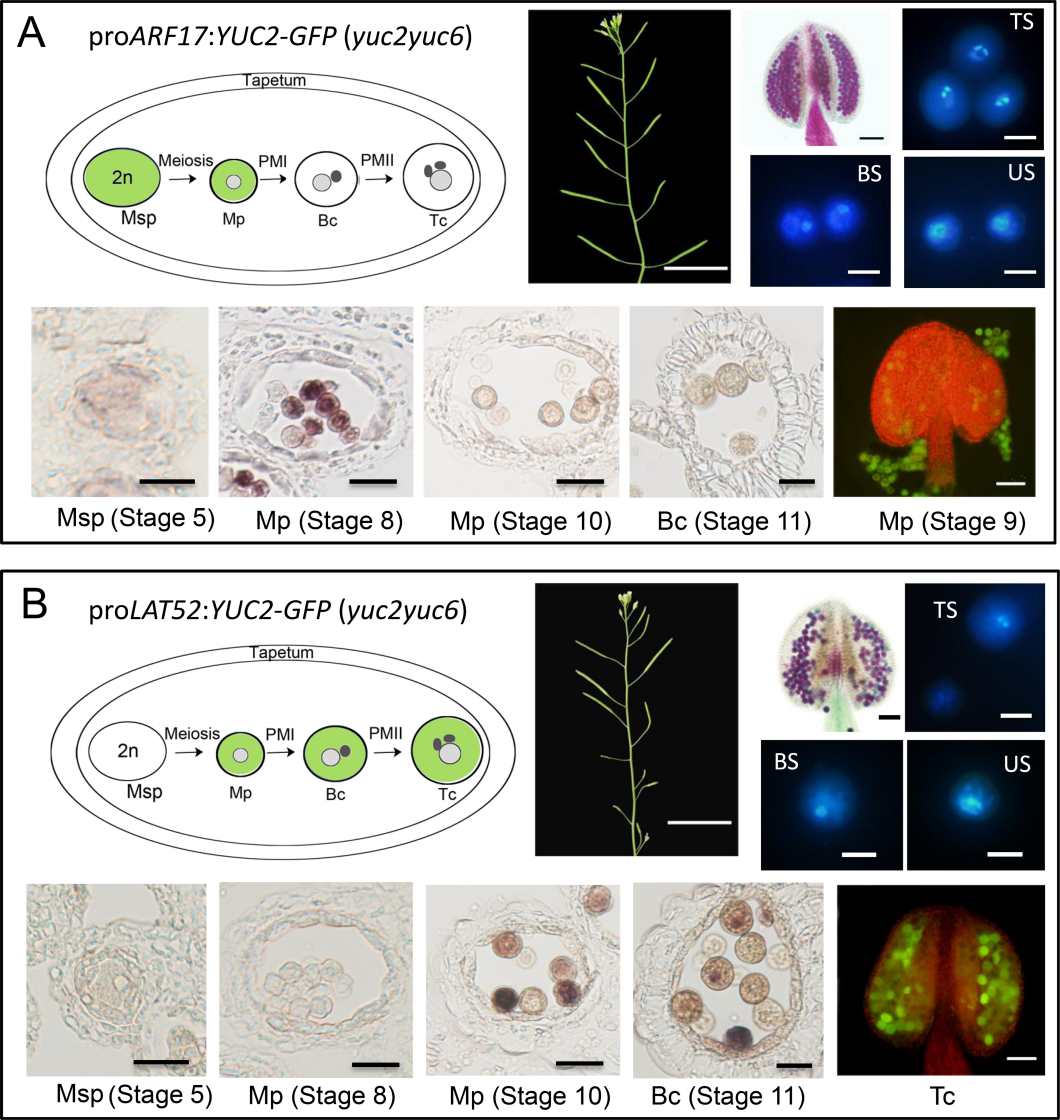
Localized auxin production in microsporocytes and microspores rescued the pollen defects in *yuc2yuc6* mutants. The green color indicates the expression pattern of the *ARF17* (A) and *LAT52* (B) promoter. The *ARF17* promoter is known to be active in microsporocytes and microspores [63]. *LAT52* is expressed in microspore and gametophyte specifically. Msp, Microsporocytes; Mp, microspores; Bc, Bicellular pollen; Tc, Tricellular pollen. Morphology of adult shoots (Bars = 2 cm), Alexander staining (Bars = 100 μm) of anthers and DAPI staining (Bars = 10 μm) of pollens from Pro*ARF17*:*YUC2-GFP* (*yuc2yuc6*) (A) and Pro*LAT52*:*YUC2-GFP* (*yuc2yuc6*) (B) transgenic plants. Note that *YUC2-GFP* expression driven by *ARF17* and *LAT52* promoters are sufficient to rescue the *yuc2yuc6* defects. In situ hybridization of GFP (Bars = 20 μm) and fluorescence images (Bars = 100 μm) of the YUC2-GFP fusion protein in anthers from *yuc2yuc6* transformed with Pro*ARF17*:*YUC2-GFP* (A) and Pro*LAT52*:*YUC2-GFP* (B). In Pro*ARF17*:*YUC2-GFP* (*yuc2yuc6*) anther, GFP is expressed in microsporocytes and in microspores at early stage (A). In Pro*LAT52*:*YUC2-GFP* (*yuc2yuc6*) anther, GFP is significantly expressed in microspores at late stage and in bicellular pollens (B). TS, Tricellular Stage; BS, Bicellular Stage; US; Unicellular Stage.

We next used promoters specific for microsporocytes and microspores (pro*ARF17*) [63] to drive the expression of *YUC2-GFP* in *yuc2yuc6* plants (Fig 6). All of the 5 independent T1 pro*ARF17*:*YUC2-GFP* (*yuc2yuc6*) lines showed almost complete rescue of the sterile phenotypes of *yuc2yuc6* (Fig 6A). In addition, the pollen defects were fully rescued in the transgenic plants (Fig 6A and S5 Fig). In the transgenic plants, *YUC2-GFP* expression was observed in microsporocytes and was significant in early stages of microspores (Fig 6A). Fig 6A also showed that the YUC2-GFP protein accumulated in early stages of microspores. Because *ARF17* promoter drives gene expression in both microsporocytes and microspores, we further tested whether localized auxin biosynthesis in microspores is sufficient to rescue *yuc2yuc6*. We used the promoter pro*LAT52*, which is specifically activated in male gametophyte [14, 64], to drive the expression of *YUC2-GFP* in *yuc2yuc6* plants (Fig 6B). All of the 6 independent T1 pro*Lat52*:*YUC2-GFP* (*yuc2yuc6*) lines showed partial rescue of the fertility defects of *yuc2yuc6* (Fig 6B). The pro*Lat52*:*YUC2-GFP* (*yuc2yuc6*) plants were fertile at a late reproductive development stage (Fig 6B). During late reproduction development, 30% to 70% of the pollen in the transgenic lines appeared normal in the anthers (Fig 6B and S5 Fig). At bicellular stage, 70.7% of the unicellular pollen can develop into bicellular pollen, and 18% of the microspores are arrested at unicellular stage. At tricellular stage, pro*Lat52*:*YUC2-GFP* (*yuc2yuc6*) produced 56.9% tricellular pollens, and 31.8% pollens were aborted (Fig 6B and S5 Fig). These results indicated that pro*Lat52*:*YUC2-GFP* could partially support the microspores development past PMI and PMII. In the *proLat52*:*YUC2-GFP* (*yuc2yuc6*) transgenic plants, *YUC2-GFP* was transcribed from late stages of microspores (Fig 6B). Meanwhile, YUC2-GFP protein was detected in pollen at stage 13 (Fig 6B). The observation that auxin produced in microspores partially rescued the sterile phenotype of *yuc2yuc6* suggested that early stages of pollen development including PMI of unicellular microspores require auxin. The sterility rescue efficiencies of pro*ARF17*:*YUC2-GFP* (*yuc2yuc6*) and pro*YUC2*:*YUC2-GFP (yuc2yuc6)* (S3 Fig) were significantly higher than that in pro*Lat52*:*YUC2-GFP* (*yuc2yuc6*) transgenic plants. We noticed that the expression of *YUC2-G*FP or *GFP* was at earlier stage of microspores in pro*ARF17*:*YUC2-GFP* (*yuc2yuc6*) and in pro*YUC2*:*GFP* (S2 Fig) than that in pro*Lat52*:*YUC2-GFP* (*yuc2yuc6*) transgenic plants (Fig 6). Therefore, it is likely that auxin may be required at early phase of microspore development. Combined with the genetic data that *YUC2* and *YUC6* act as sporophytic genes, we conclude that the auxin synthesized in the sporophytic microsporocytes is essential for early stages of pollen development in plants. Therefore, auxin produced in sporophyte contributes to male gametophyte during the generation alternation in plant.

## Discussion

Both *YUC2* and *YUC6* are known required for pollen development and the *yuc2yuc6* double mutants are male sterile. Here we further defined that the male sterility of *yuc2yuc6* is caused by defects in early stages of pollen development including the first mitotic cell division (PMI) of microspores. Moreover, we show that early stages of microspore development and PMI require auxin produced in the diploid sporophytic microsporocytes, indicating that sporophytic cells not only provide nutrients and cell wall materials for pollen development, but also supply hormone and signaling molecules to haploid cells. Our results also demonstrate that different sporophytic cells play different roles in male gametophytic development. Tapetum cells provide nutrients, but auxin produced in tapetum cells is not sufficient to support early stages of pollen development. In contrast, auxin synthesized in sporophytic microsporocytes is necessary and sufficient for male gametophytic development.

Because *yuc2yuc6* double mutants could undergo meiosis successfully and our results showed that supply of auxin after meiosis could partially rescue the pollen defects of *yuc2yuc6* (Fig 6, *ProLat52*:*YUC2-GFP* (*yuc2yuc6*)), we conclude that auxin produced by YUC2 and YUC6 may not be required for male meiosis. Following meiosis, the microspore undergoes two rounds of mitosis to form mature pollen during anther development. PMI is the first round of mitosis for pollen formation. Several lines of evidence show that auxin produced by YUCs is essential for PMI. We showed that YUC2 and YUC6 are the main enzymes responsible for auxin synthesis in anther based on the expression of the auxin reporter *DR5*:*GFP/GUS* (Fig 1 and S1 Fig). Our results are consistent with previous studies showing DR5 activity in anthers possibly resulting from auxin synthesis [54]. Secondly, we found that the microspores of y*uc2yuc6* were aborted and degenerated before PMI and failed to form mature pollen (Fig 3 and Fig 4). Consistent with this phenotype, we found significant AUX/IAA degradation, which is presumably induced by auxin, in unicellular microspores judging from the expression of the modified DII-Venus reporter (Fig 2). Lastly, we revealed that *YUC2-GFP* expressed in microspores at early gametophyte stages could partially rescue the *yuc2yuc6* pollen defect (Fig 6, *ProLat52*:*YUC2-GFP* (*yuc2yuc6*)). These results demonstrated that auxin is required for early stages of pollen development. Because most of the microspores in *yuc2yuc6* were arrested prior to PMI, we could not exclude the possibility that auxin is also required in subsequent steps such as PMII. Auxin regulates plant development mainly through auxin response factors (ARFs). Among the *ARF* genes, *ARF6*, *ARF8*, *ARF10*, *ARF16*, and *ARF17* are expressed in unicellular microspores [65]. The *arf17* mutant shows defective pollen formation [63], suggesting that auxin synthesized by YUCs may control male gametophyte development through ARF17. However, we noticed that pollen development defects in *arf17* and *yuc2yuc6* were not the same. The *arf17* mutants displayed defective pollen coat formation whereas it was not the case for *yuc2yuc6*. Therefore, we believe that ARF17 may have unique functions in the earlier stages of pollen development that do not overlap with those of YUC2 and YUC6, such as controlling of the morphogenesis of pollen wall.

It was reported that pollen development in *tir1 afb1 afb2 afb3* quadruple mutants was not significantly compromised, an observation that was quite different from the severe pollen defects found in *yuc2yuc6*. It is puzzling that disruption of auxin signaling and auxin biosynthesis resulted in different phenotypes. It was known that some of the *tir1 afb1 afb2 afb3* plants can survive and produce seeds, but auxin transport mutants such as *pin1* and auxin biosynthesis mutants including *yuc1yuc4* were completely sterile [49, 66]. It is likely that other AFB proteins such as AFB4 and AFB5 may compensate the loss of *tir1 afb1 afb2 afb3*. In addition, we noticed that *afb1* and *afb3* in the quadruple mutants still produced truncated transcripts and might have produced residual protein activities [67]. The fact that *tir1 afb1 afb2 afb3* were not a complete null in auxin receptor may account for the observed paradoxical results. AFB5 is expressed in bicellular pollens (S1 Table). Analysis of higher order *tir1 afb* mutants may clarify why auxin signaling mutants behaved differently from auxin biosynthesis and transport mutants.

Most of the known functions of auxin in flowering plants are related to sporophyte generation, which is the dominant generation. The auxin pathways are evolutionary conserved in the plant kingdom. The homologs of auxin biosynthetic genes such as *TAAs* and *YUCs* are also used for auxin biosynthesis in moss *Physcomitrella patens* and Liverwort *Marchantia polymorpha* [68, 69], whose haploid gametophyte is the dominant generation. In liverwort, auxin synthesized by YUCs is essential for normal gametophyte development and dormancy [69], implying that auxin may also play essential roles in gametophyte generation in flowering plants. However, it is difficult to study the roles of auxin in male gametophyte development because male gametophyte has been reduced to several cells within the diploid sporophyte. Our detailed analysis of the auxin biosynthetic mutants *yuc2yuc6* revealed that auxin is required for male gametophyte development.

More importantly, we show that auxin produced in microsporocytes, which are sporophytic cells, is necessary for normal progression of the haploid microspores. Our genetic data suggest that *YUC2* and *YUC6* affect early stages of pollen development mainly via a sporophytic effect, implying that auxin required for pollen development may be synthesized by YUC2 and YUC6 in sporophytic anther tissues. These data are consistent with the transcription patterns of *YUC2* and *YUC6* in microsporocytes, microspores and anther somatic cell layers at premeiotic and meiotic stages [54]. In anther, tapetum cells and microsporocytes are closely related sporophyte cells involved in microspore/male gametophyte development. It was proposed that auxin could be transported to developing pollen from tapetum cells [56]. However, the expression of *YUC2* in the tapetum cell of *yuc2yuc6* could not lead to viable pollen (Fig 5 and S4 Fig), suggesting that the auxin from tapetum is not sufficient to support pollen development. On the other hand, the expression of YUC2-GFP in microsporocytes completely rescued the sterility phenotypes of *yuc2yuc6* (Fig 6), demonstrating that auxin synthesized in microsporocytes is sufficient for pollen development.

We propose that auxin production in diploid microsporocytes is necessary and sufficient for the early stages of the development of the haploid cells during pollen development. Our conclusion is mainly based on two observations: 1) *YUC2* and *YUC6* are sporophytic genes and the *yuc2yuc6* fail to produce viable pollen; 2) expression of *YUC2* driven by specific promoters rescued *yuc2yuc6* (Fig 6). The *ARF17* promoter is active in both microsporocytes (sporophytic) and microspores (gametophytic) (Fig 6). Therefore, it is conceivable that the rescued *yuc2yuc6* phenotypes by *YUC2-GFP* driven by *ARF17* promoter were caused by ectopic auxin production in the haploid microspores. However, from our genetic analysis, it is clear that viable and fully functional pollen that lacks *YUC2* and *YUC6* can be produced from *yuc2^-/-^yuc6^+/-^* or *yuc2^+/-^yuc6^-/-^*, demonstrating that auxin does not have to be produced in microspores for the development of viable pollen. Rather auxin produced by YUCs in sporophytic cells (microsporocytes to be exact) is sufficient to guide the progressive development of microspores.

Microsporocytes developing into mature pollen through PMI and PMII is a continuing process. Production of auxin in microsporocytes is an efficient way to regulate newly formed microspores to undergo PMI and other processes of early male gametophyte development. Using two different diploid cell types (tapetum and microsporocytes) to provide nutrients and hormonal signals for male gametophyte development may also be advantageous in terms of efficiency and specificity.

## Material and methods

### Plant materials and growth conditions

All plants used in this study were in the Columbia-0 genetic background. The *yuc2yuc6* mutants have been described previously [49]. All relevant primer sequences were listed in S2 Table. The DII-VENUS (N799173) and mDII-VENUS (N799174) seeds were ordered from the European Arabidopsis Stock Centre (NASC). Plants were grown under long-day conditions (16 hr light/8 hr dark) in a ~22°C growth room. The *DR5*:*GFP* and *DR5*:*GUS* reporter lines were introduced into *yuc2yuc6* by genetic cross.

### Microscopy

Plants were photographed using a Nikon digital camera (D-7000). Alexander solution was prepared as previously described [70]. Anthers were dissected and immersed in Alexander solution for 0.5 hr, and images were obtained under a microscope with an Olympus BX51 digital camera (Olympus, Japan). Plant materials for the semi-thin sections were prepared and embedded in Spurr resin as described in [25] and cut into 1-μm thick sections, stained with toluidine blue, then photographed with an Olympus BX51 digital camera. For transmission electron microscopic analysis, the same-stage anthers of wild type (WT) and *yuc2yuc6* were fixed and embedded as previously described [25]. Green fluorescent protein (GFP) fluorescence in Pro*ARF17*:*YUC2-GFP* (*yuc2yuc6*), Pro*LAT52*:*YUC2-GFP* (*yuc2yuc6*), Pro*MSP1*:*DII*, and Pro*MSP1*:*mDII* transgenic plants was detected under a fluorescence microscope (Olympus BX51). GFP fluorescence in Pro*A9*:*YUC2-GFP* (*yuc2yuc6*) transgenic plants and WT or *yuc2yuc6* expressing *DR5*:*GFP* was detected by confocal laser scanning microscopy (Carl Zeiss, LSM 5 PASCAL).

### Plasmid construction and plant transformation

To generate *YUC2-GFP*, we amplified *YUC2* cDNA without the stop codon from inflorescence mRNA by standard RT-PCR (see S2 Table for primers) for cloning into the modified pCAMBIA1300 binary vector (CAMBIA, Australia), pCAMBIA1300-*GFP*. The resulting construct was named pCAMBIA1300-*YUC2-GFP*. The promoter sequences of *YUC2*, *YUC6*, *ARF17*, *ATA7* and *A9* were PCR-amplified from genomic DNA of WT Col-0. The promoter sequence of *LAT52* was PCR-amplified from genomic DNA of tomato. The primers for amplification were listed in S2 Table. The amplified sequences were first inserted into pMD19-T (Takara) for sequence verification, then subcloned into the vector pCAMBIA1300-*YUC2-GFP* for plant transformation. The promoter sequences for *YUC2* and *YUC6* were subcloned into pCAMBIA1300-*GFP* for plant transformation. The promoter sequence for *MSP1* was PCR-amplified from genomic DNA of WT Col-0, then subcloned into the vector pCAMBIA1300 to obtain pCAMBIA1300-*proMSP1*. The DII-VENUS and mDII-VENUS sequences were amplified from genomic DNA from DII-VENUS and mDII-VENUS plants. The amplified sequences were inserted into pMD19-T (Takara) for verification, then subcloned into the vector pCAMBIA1300-*proMSP1* for plant transformation. For plant transformation, all plasmids were introduced into the Agrobacterium strain GV3101 and transformed into plants by the floral dip method[71]. For tissue specific rescue experiments, all of the constructs were transformed into the offspring of *yuc2^-/-^yuc6^+/-^*. After collected the T1 seeds, we first selected transgenic plants by growing on MS media containing hygromycin. We then genotyped the transgenic plants for *yuc2yuc6* double mutants[49].

### In situ hybridization

The probe fragments were amplified from plasmid containing *GFP* (pCAMBIA1300-*GFP*) with primers GFP-F and GFP-R. The PCR products were cloned into the pBluescriptSK vector and confirmed by sequencing. Plasmid DNA was digested with HindIII or BamHI. The digestion products were used as templates for transcription into sense and antisense probes by T3 and T7 RNA polymerases, respectively (Roche). Oligonucleotide sequences of GFP-F and GFP-R are provided in S2 Table. Images were taken using the Olympus BX-51 microscope.

### RNA extraction and real-time RT-PCR

Total RNA was extracted from the inflorescences of T1 transgenic plants by the Trizol method (Invitrogen, USA) following the manufacturer’s instructions. Quantitative real-time PCR involved an ABI PRISM 7300 detection system (Applied Biosystems, USA) with SYBR Green I master mix (Toyobo, Japan). Relevant primer sequences are in S2 Table. β-Tubulin was as a constitutive expression control. Three biological repeats were used for gene expression analysis.

## Supporting information

S1 Fig Disruption of *YUC2* and *YUC6* completely abolishes the expression of the auxin reporter *DR5*:*GUS* in the flowers. (A) Expression of the *DR5*:*GFP* auxin-responsive reporter in *yuc2* and *yuc6* (Bars = 100 μm). (B) Expression of the *DR5*:*GUS* auxin-responsive reporter in wild type, *yuc2*, *yuc6* and *yuc2yuc6* flowers. Note that the GUS staining signal of DR5:GUS disappeared in the *yuc2yuc6* mutant.
(TIF)

S2 Fig The expression patterns of *ProYUC2*:*GFP* and *ProYUC6*:*GFP* reporters during pollen development. Fluorescence images of the pro*YUC2*:*GFP* (A-G) and pro*YUC6*:*GFP* (H-N). In situ hybridization of GFP in pro*YUC2*:*GFP* transgenic plants (O). Bars = 50 μm for the anther in I. Bars = 10 μm for all the other images.
(TIF)

S3 Fig Genetic complementation of *yuc2yuc6* double mutants by expressing the *YUC2 cDNA* fused with GFP at its C-terminus. (A) Construct of the Pro*YUC2*:*YUC2-GFP* plasmid. N, nopaline synthase terminator. (B**)** Morphology of adult shoots (Bars = 2cm) from *yuc2yuc6* and *yuc2yuc6* complemented with a *YUC2-GFP* under the control of the *YUC2* promoter (Pro*YUC2*:*YUC2-GFP*). The Pro*YUC2*:*YUC2-GFP* can completely rescue the sterility phenotype of *yuc2yuc6*. (C and D) Alexander staining (Bars = 100 μm) (C) and DAPI staining (Bars = 10 μm) (D) of Pro*YUC2*:*YUC2-GFP* (*yuc2yuc6*) anthers and pollens. The pollen defects are rescued in Pro*YUC2*:*YUC2-GFP* (*yuc2yuc6*) plants.
(TIF)

S4 Fig *YUC2* expression driven by *ATA7* promoters did not rescue the *yuc2yuc6* defects. The green color indicates the expression pattern of the *ATA7* promoter. Msp, Microsporocytes; Mp, microspores; Bc, Bicellular pollen; Tc, Tricellular pollen. Morphology of adult shoots (Bars = 2cm). Alexander staining (Bars = 100 μm) and DAPI staining (Bars = 10 μm) of Pro*ATA7*:*YUC2-GFP* (*yuc2yuc6*) anthers and pollens. Note that the sterility phenotype and pollen defects were not rescued in Pro*ATA7*:*YUC2-GFP* (*yuc2yuc6*) transgenic plants. TS, Tricellular Stage; BS, Bicellular Stage; US; Unicellular Stage. Fluorescence images (Bars = 100 μm) showed that YUC2-GFP fusion protein was not observed in transgenic plants anthers. In situ hybridization of GFP (Bars = 20 μm) showed that GFP is weakly expressed in tapetum cell at stage 8 in Pro*ATA7*:*YUC2-GFP* (*yuc2yuc6*) anther.
(TIF)

S5 Fig DAPI staining and real-time quantitative RT-PCR analysis of WT and transgenic plants. (A)Quantitative analysis of pollen defects of transgenic plants (n>500 for each stage for each type of plants). Uc, Unicellular pollen; Bc, Bicellular pollen; Tc, Tricellular pollen; De, Degenerated pollen. (B) cDNA sample from the inflorescences of wild type Col, *yuc2yuc6* and *yuc2yuc6* transformed with Pro*ARF17*:*YUC2-GFP*, Pro*LAT52*:*YUC2-GFP*, Pro*A9-*:*YUC2-GFP* or Pro*ATA7*:*YUC2-GFP*. Data are mean±SD normalized to *TUBULIN* and compared with Col from three biological replicates. The transcript levels of *YUC2* in all the transgenic plants were equal to or higher than that in Col.
(TIF)

S1 Table The transcription data of the TIR/AFB family members during pollen development. These data are extracted from a published paper [65]. MS, microspores; BCP, bicellular pollen; TCP, tricellular pollen; MPG, mature pollen.
(XLSX)

S2 Table Primers used in this study. (XLSX)
