# Supplementary figures and images for: Auxin production in diploid microsporocytes is necessary and sufficient for early stages of pollen development
Source: PLoS Genet. 2018 May 29;14(5):e1007397. doi: 10.1371/journal.pgen.1007397 (PMC5993292; doi:10.1371/journal.pgen.1007397)

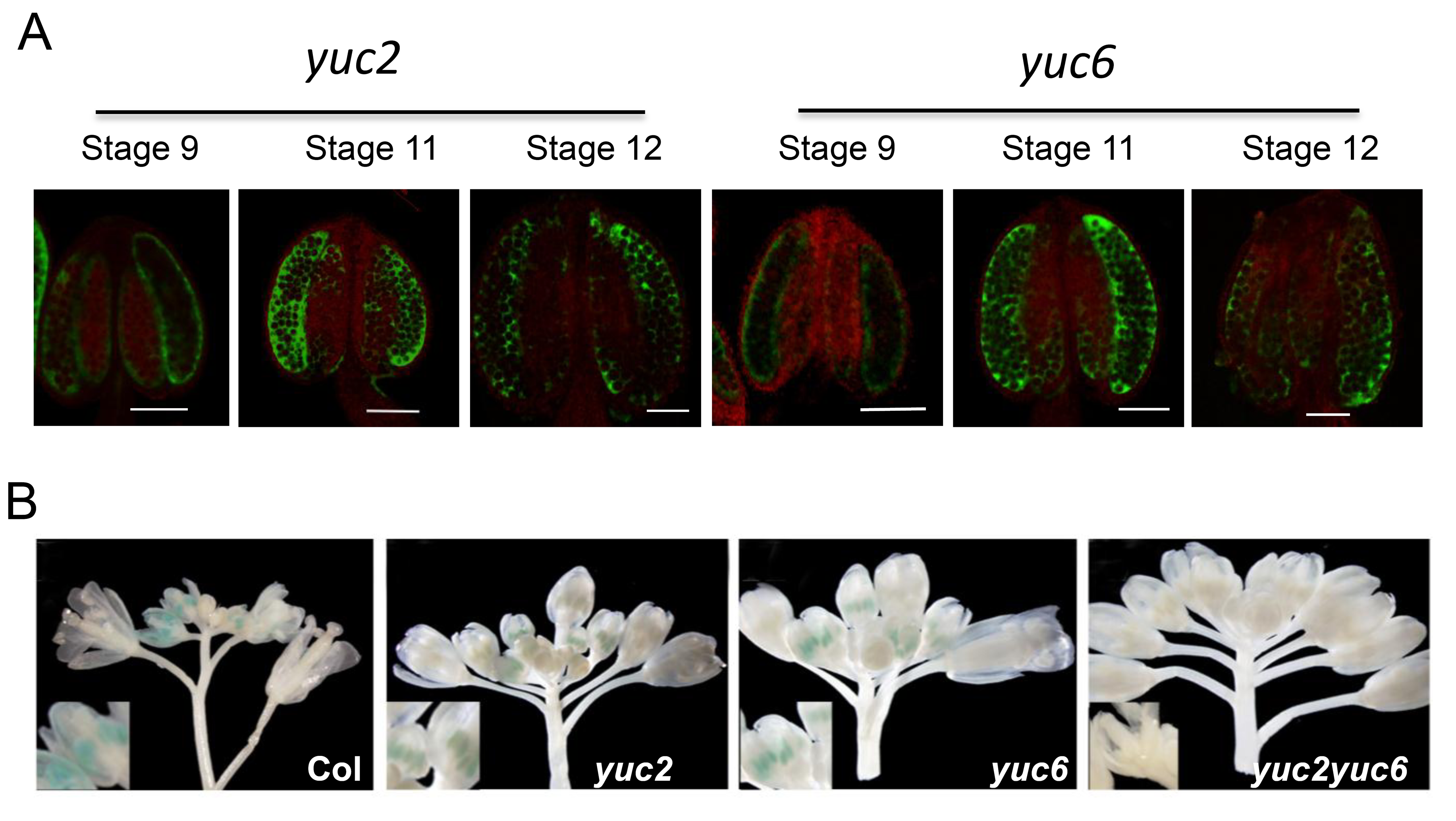

Supplement: S1 Fig — (A) Expression of the DR5:GFP auxin-responsive reporter in yuc2 and yuc6 (Bars = 100 μm). (B) Expression of the DR5:GUS auxin-responsive reporter in wild type, yuc2, yuc6 and yuc2yuc6 flowers. Note that the GUS staining signal of DR5:GUS disappeared in the yuc2yuc6 mutant. (TIF) [file pgen.1007397.s001.tif]

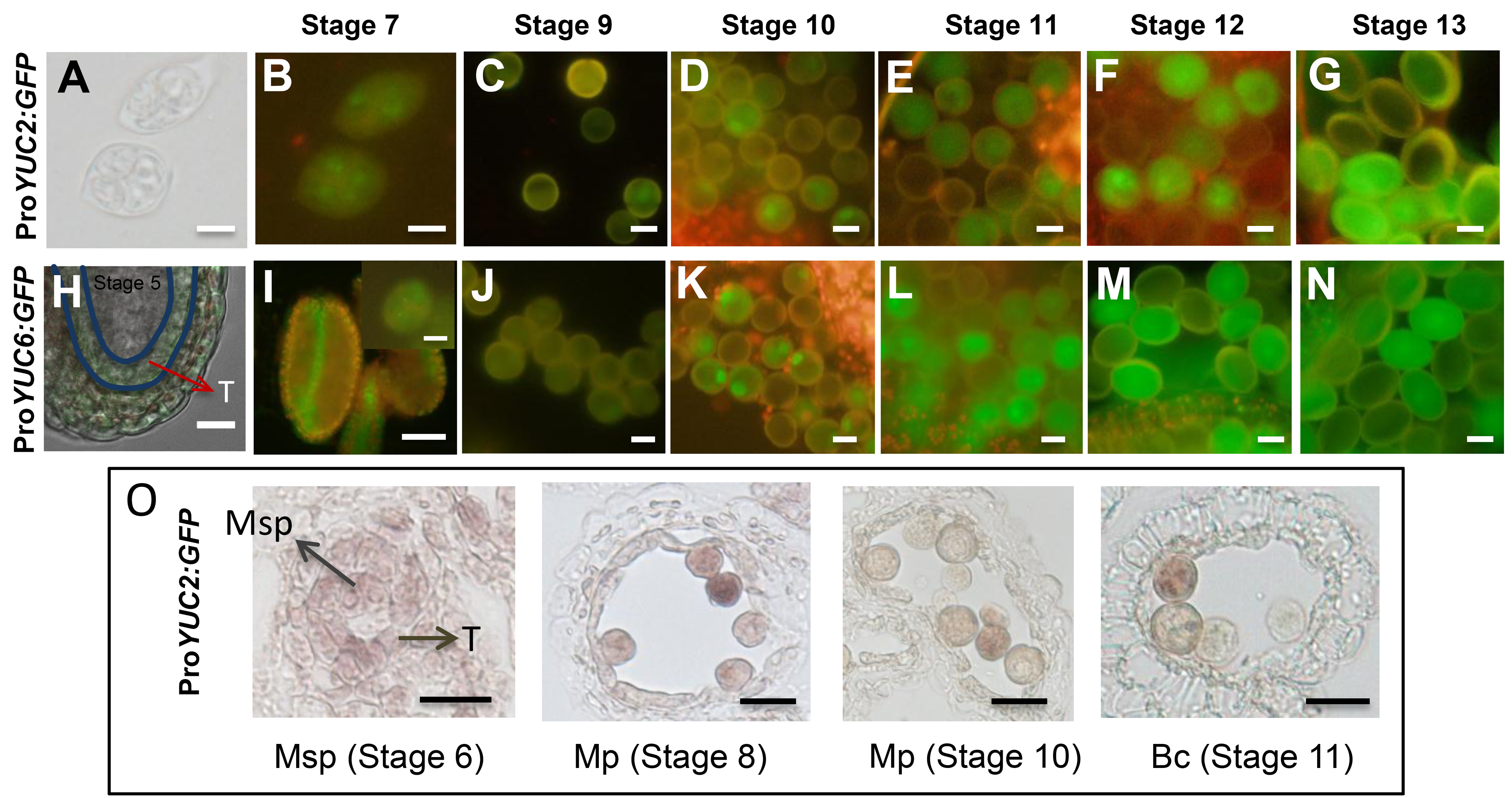

Supplement: S2 Fig — Fluorescence images of the proYUC2:GFP (A-G) and proYUC6:GFP (H-N). In situ hybridization of GFP in proYUC2:GFP transgenic plants (O). Bars = 50 μm for the anther in I. Bars = 10 μm for all the other images. (TIF) [file pgen.1007397.s002.tif]

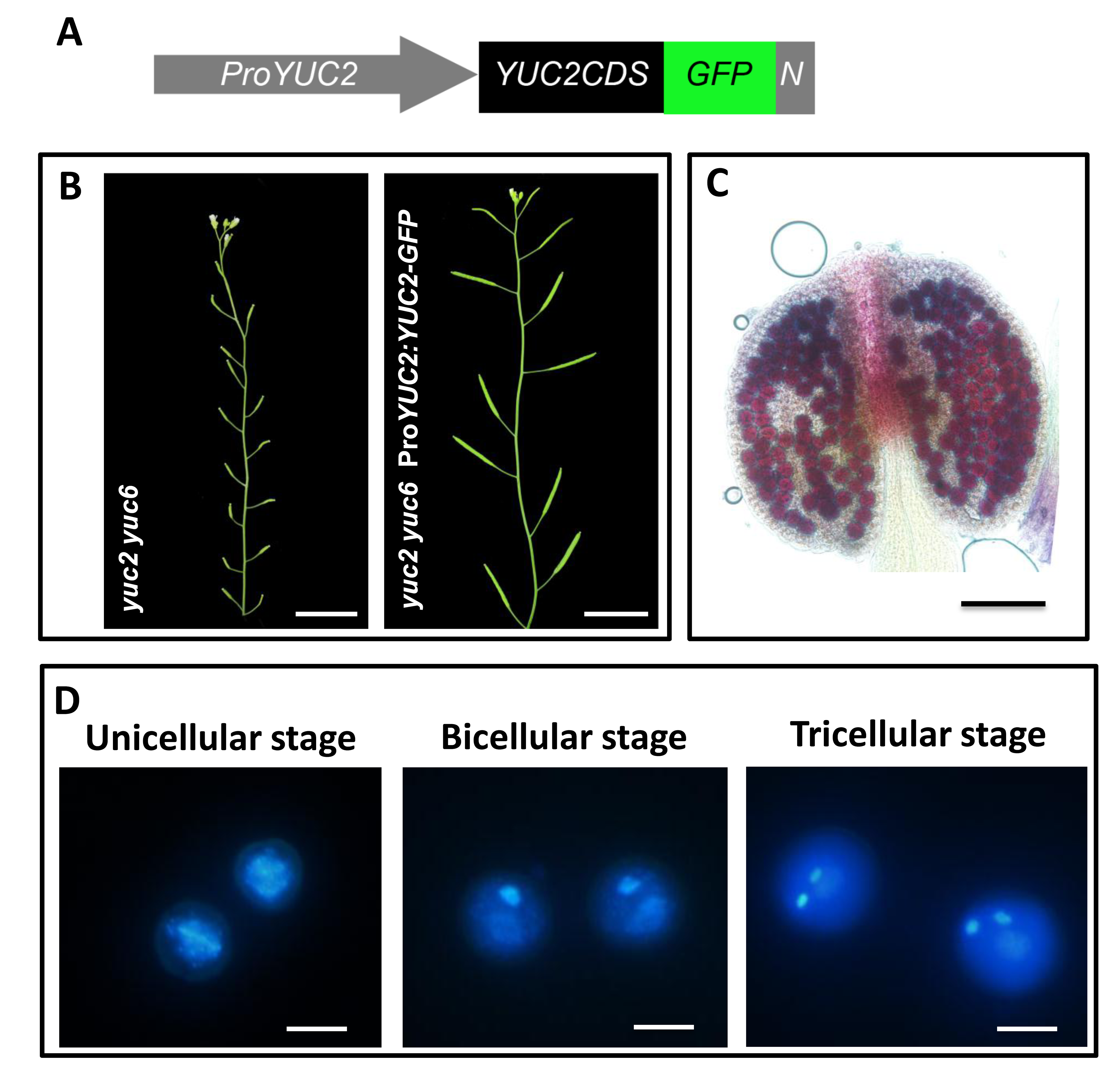

Supplement: S3 Fig — (A) Construct of the ProYUC2:YUC2-GFP plasmid. N, nopaline synthase terminator. (B) Morphology of adult shoots (Bars = 2cm) from yuc2yuc6 and yuc2yuc6 complemented with a YUC2-GFP under the control of the YUC2 promoter (ProYUC2:YUC2-GFP). The ProYUC2:YUC2-GFP can completely rescue the sterility phenotype of yuc2yuc6. (C and D) Alexander staining (Bars = 100 μm) (C) and DAPI staining (Bars = 10 μm) (D) of ProYUC2:YUC2-GFP (yuc2yuc6) anthers and pollens. The pollen defects are rescued in ProYUC2:YUC2-GFP (yuc2yuc6) plants. (TIF) [file pgen.1007397.s003.tif]

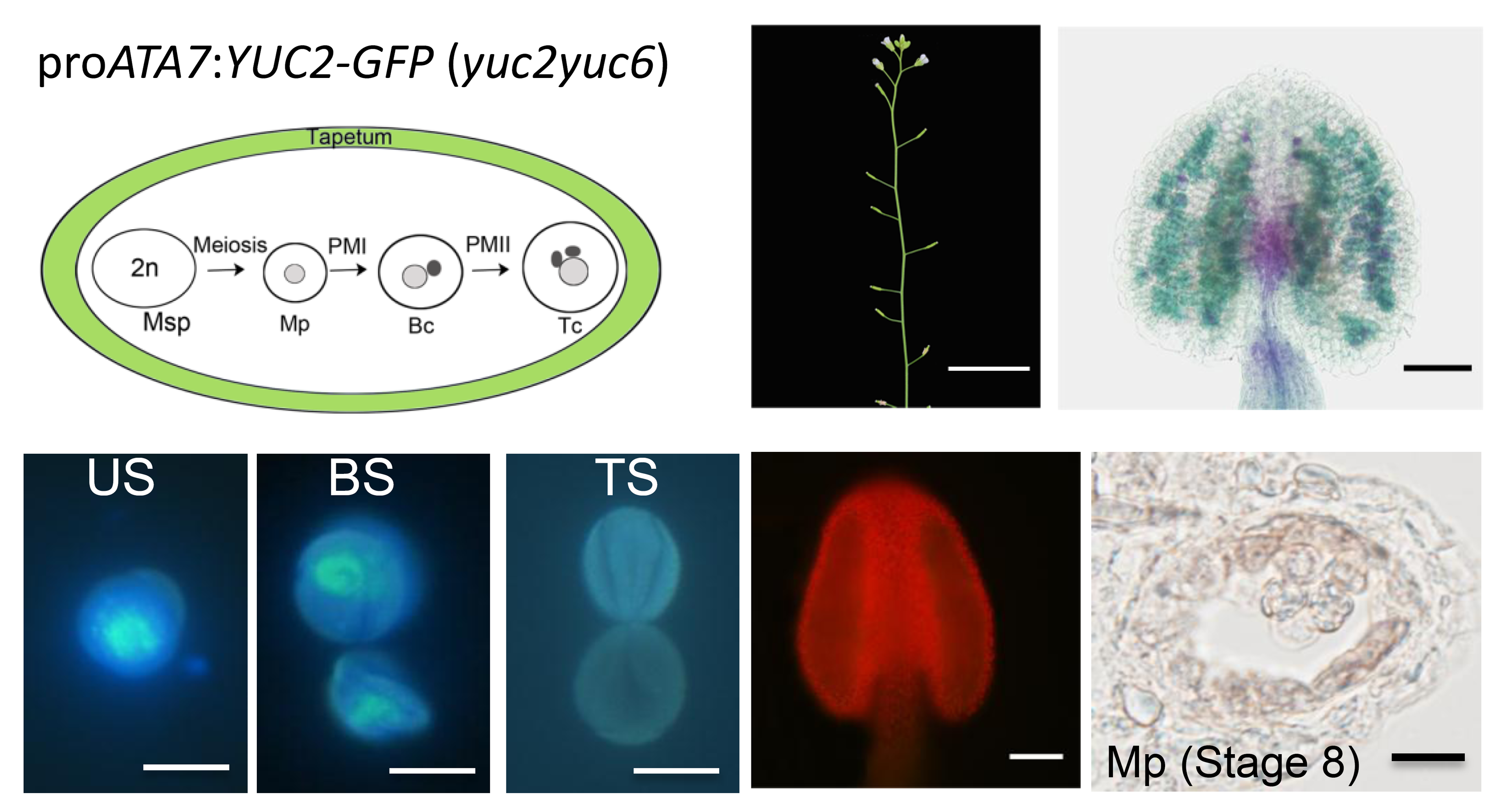

Supplement: S4 Fig — The green color indicates the expression pattern of the ATA7 promoter. Msp, Microsporocytes; Mp, microspores; Bc, Bicellular pollen; Tc, Tricellular pollen. Morphology of adult shoots (Bars = 2cm). Alexander staining (Bars = 100 μm) and DAPI staining (Bars = 10 μm) of ProATA7:YUC2-GFP (yuc2yuc6) anthers and pollens. Note that the sterility phenotype and pollen defects were not rescued in ProATA7:YUC2-GFP (yuc2yuc6) transgenic plants. TS, Tricellular Stage; BS, Bicellular Stage; US; Unicellular Stage. Fluorescence images (Bars = 100 μm) showed that YUC2-GFP fusion protein was not observed in transgenic plants anthers. In situ hybridization of GFP (Bars = 20 μm) showed that GFP is weakly expressed in tapetum cell at stage 8 in ProATA7:YUC2-GFP (yuc2yuc6) anther. (TIF) [file pgen.1007397.s004.tif]

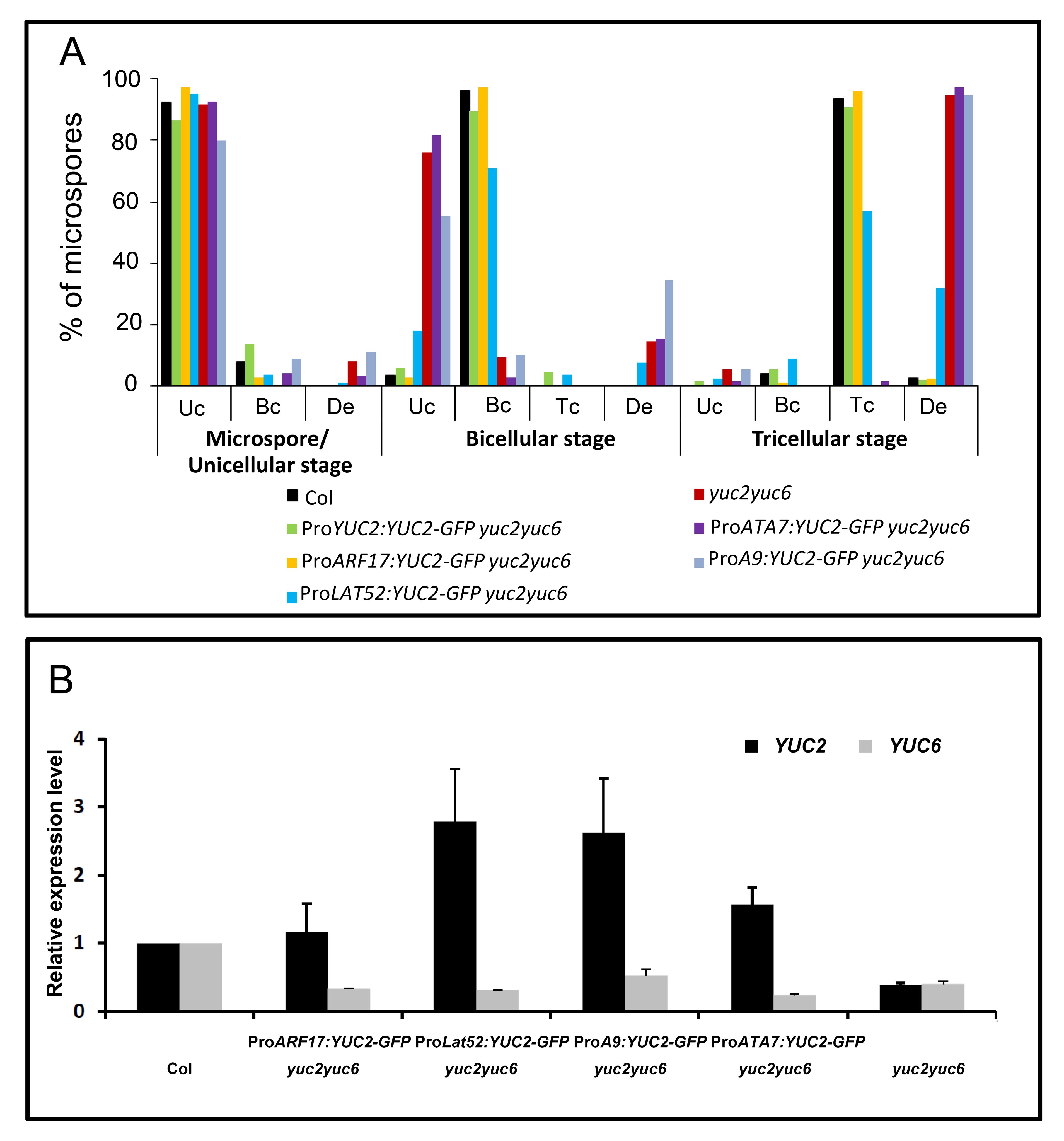

Supplement: S5 Fig — (A)Quantitative analysis of pollen defects of transgenic plants (n>500 for each stage for each type of plants). Uc, Unicellular pollen; Bc, Bicellular pollen; Tc, Tricellular pollen; De, Degenerated pollen. (B) cDNA sample from the inflorescences of wild type Col, yuc2yuc6 and yuc2yuc6 transformed with ProARF17:YUC2-GFP, ProLAT52:YUC2-GFP, ProA9-:YUC2-GFP or ProATA7:YUC2-GFP. Data are mean±SD normalized to TUBULIN and compared with Col from three biological replicates. The transcript levels of YUC2 in all the transgenic plants were equal to or higher than that in Col. (TIF) [file pgen.1007397.s005.tif]
